# Emergency Department Visits by Patients with Mental Health Disorders — North Carolina, 2008–2010

**Published:** 2013-06-14

**Authors:** Anne M. Hakenewerth, Judith E. Tintinalli, Anna E. Waller, Amy Ising, Tracy DeSelm

**Affiliations:** Texas Cancer Registry, Texas Dept of State Health Svcs; Carolina Center for Health Informatics, Dept of Emergency Medicine, Univ of North Carolina at Chapel Hill

Patients with mental health disorders (MHDs) use the emergency department (ED) for acute psychiatric emergencies, for injuries and illnesses complicated by or related to their MHD, or when psychiatric or primary-care options are inaccessible or unavailable (1,2). An estimated 5% of ambulatory-care visits in the United States during 2007–2008 were made by patients with primary mental health diagnoses (3). To measure the incidence of ED visits in North Carolina with MHD diagnostic codes (MHD-DCs), the Carolina Center for Health Informatics (University of North Carolina at Chapel Hill) analyzed ED visits occurring during the period 2008–2010 captured by the North Carolina Disease Event Tracking and Epidemiologic Collection Tool (NC DETECT). This report describes the results of that analysis, which indicated that nearly 10% of ED visits had one or more MHD-DCs assigned to the visit and the rate of MHD-DC-related ED visits increased seven times as much as the overall rate of ED visits in North Carolina during the study period. Those with an MHD-DC were admitted to the hospital from the ED more than twice as often as those without MHD-DCs. Stress, anxiety, and depression were diagnosed in 61% of MHD-DC-related ED visits. The annual rate of MHD-DC-related ED visits for those aged ≥65 years was nearly twice the rate of those aged 25–64 years; half of those aged ≥65 years with MHD-DCs were admitted to the hospital from the ED. Mental health is an important component of public health (4). Surveillance is needed to describe trends in ED use for MHDs to develop strategies to prevent hospitalization, improve access to ambulatory care, and develop new ways to provide ED care for the elderly with MHDs.

ED visit data for the period 2008–2010 were extracted from NC DETECT, a population-based, statewide public health surveillance system that contains ED visit data (5,6) for 99% of ED visits in North Carolina occurring during the study period. ED visits were characterized by sex and age group, ED disposition, and type of MHD. MHD-DCs were identified from the *International Classification of Diseases, Ninth Revision, Clinical Modification* (ICD-9-CM) codes for mental disorders (290–299); symptoms, signs, and ill-defined conditions (787–789.9); and supplementary codes (V11–79). ICD-9-CM codes for poisoning and overdose, metabolic or structural encephalopathies that are classified as psychiatric diagnostic codes by ICD-9-CM, substance abuse disorders, and tobacco use disorder were excluded. For each ED visit, a mental health ICD-9-CM diagnostic code in any one of up to 11 positions classified that visit as MHD-DC-related. Visit records with more than one MHD-DC were counted as a single MHD-DC-related visit. Using the first-listed MHD-DC for the ED visit, MHDs were subcategorized into 11 groups of clinically similar diagnostic categories for calculating rates. For purposes of regression analyses, all MHD-DCs were classified as present or absent for each ED visit. Data were extracted and stratified for univariate and two-way descriptive analyses, and annual rates were calculated per 10,000 population. Risk ratios were computed using log binomial regression with Poisson robust variances.

From 2008 to 2010, the annual number of ED visits in North Carolina increased by 5.1%, from 4,190,911 to 4,405,676, and MHD-DC-related ED visits increased by 17.7%, from 347,806 to 409,276 (Table 1). By 2010, ED visits with MHD-DCs accounted for 9.3% of all ED visits; 31.1% of ED visits with MHC-DCs resulted in hospital admission, compared with 14.1% of all ED visits.

For each ED visit, up to 11 diagnostic codes are captured by NC DETECT. One quarter of first-listed MHD-DCs were in the first-listed diagnostic code position, 56% of the MHD-DCs were within the first three diagnostic code positions, and 77% were within the first five. “Stress/Anxiety/Depressive disorders” was the MHD-DC category with the highest number of ED visits (Table 2).

Increasing age was associated with an increase in hospital admission, with 14% of children aged <15 years admitted and 51% of adults aged ≥65 years admitted (Table 3). The highest admission proportion was for ED visits associated with dementia (60.5%) (Table 2). Population-based rates of MHD-DC related visits for those aged ≥65 years were very high for any MHD diagnosis compared with all other age groups, driven primarily by higher rates of schizophrenia/delusions/psychoses, dementia, and stress/anxiety/depression (Table 4).

## Editorial Note

The ED is an important link between outpatient and inpatient services for the care of patients with MHDs. ED visits by patients with MHD-DCs are increasing more rapidly than general ED visits (3,7). Only minor changes in ICD-9-CM codes have been issued since October 2000 (8), so coding procedures for MHD likely did not change greatly during the course of the study. In this study, population-based rates of MHD-DC-related ED visits in North Carolina increased progressively from 2008 to 2010, by 14.4%, whereas the rate of all ED visits increased by only 2.1%. The rate of MHD-DC-related ED visits by patients of all ages is increasing but is especially high for those aged ≥65 years, who have the highest MHD-DC-related ED visit rate of any age group and the highest risk ratio (2.2) for hospital admission. Patients with stress/anxiety/depression accounted for the majority (60.8%) of the MHD-DC related ED visits, an unanticipated finding because such disorders often are more appropriately treated in an office setting. Hospital admissions for ED visits with MHD-DCs decreased from 35.7% in 2008 to 31.1% in 2010. The reasons for this decrease are unclear.

Good mental health services require a system of care that includes EDs, hospitals, and ambulatory-care clinics that are adequately resourced. If the trends reported in this study continue to escalate, EDs, hospitals, and (most importantly) patients will be further burdened. The high numbers of ED visits and hospital admissions for patients with any type of MHD-DCs, for those aged ≥65 years (especially with dementia), and for those with low-acuity MHDs, indicate a need for system adjustment. Strategies are needed to counteract the effects of inpatient bed shortages and the increased volume of MHD-DC-related visits to EDs. Surveillance is the first step, because identifying trends in ED use by patients with MHDs can guide policies and procedures designed to reduce hospitalization, improve access to ambulatory care services, and develop new ways to care for the elderly with MHDs in the ED.

The findings in this report are subject to at least four limitations. First, ED visit data in NC DETECT are secondary data from hospital administrative and clinical data sources; diagnostic codes typically are extrapolated by hospital coders from the patient record. Second, the percentage of ED visits identified as having associated MHD-DCs probably is an underestimate; other coding studies have reported underestimation of medical disorders when relying solely on diagnostic codes. Third, some types of ED visits by patients with MHDs, such as visits attributed to involuntary commitment or those initiated by law enforcement, likely would not be prevented by better outpatient access. Finally, coder training and experience, clinician documentation, and billing practices affect diagnosis coding for all types of medical conditions (9). For this study, MHD-DCs were categorized into clinically coherent groups by clinicians on the study team. A study reviewing ED visits for MHDs in New South Wales, Australia, using a similar classification methodology, resulted in almost identical ICD-9-CM categorization and frequencies of disorders (10).

What is already known on this topic?

The number of emergency department (ED) visits associated with mental health disorders (MHDs) is increasing in the United States. Patients with mental health disorders (MHDs) use the emergency department (ED) for acute psychiatric emergencies, for injuries and illnesses complicated by or related to their MHD, or when psychiatric or primary-care options are inaccessible or unavailable. EDs are an important part of the overall system providing health care for patients with MHDs.

What is added by this report?

In North Carolina during 2008–2010, 8.8% of ED visits were assigned at least one MHD diagnosis code (MHD-DC) among 11 possible, with a 2010 rate of 430 MHD-DC-related ED visits per 10,000 population. The rate of MHD-DC-related ED visits increased by 14.4%, whereas the rate of all ED visits increased by 2.1%, and the proportion of MHD-DC-related ED visits resulting in hospital admission was 2.3 times greater than that for all ED visits. Persons aged ≥65 years with MHD-DC-related diagnoses had the highest ED visit and admission rate of any age group.

What are the implications for public health practice?

The increasing numbers and rates of ED visits by patients with MHDs, especially the elderly, indicate a growing burden on the health-care delivery system. Standardized surveillance is needed to identify trends in ED use and the impact of any interventions.

Additional information about NC DETECT and ED visit data for North Carolina is available at http://www.ncdetect.org.

## Figures and Tables

**TABLE 1:** Number and percentage of emergency department (ED) visits related to mental health disorders (MHDs) compared with all other ED visits, overall and among those resulting in hospital admission — North Carolina, 2008–2010

Type of ED visit	2008			2009			2010		
		
	ED visits overall			ED visits overall			ED visits overall		
		
	No.	(%)	Rate per 10,000 population	No.	(%)	Rate per 10,000 population	No.	(%)	Rate per 10,000 population
MHD-related visits	347,806	(8.3)	376	381,700	(8.7)	407	409,276	(9.3)	430
All other ED visits	4,190,911	(100.0)	4,532	4,382,028	(100.0)	4,670	4,405,676	(100.0)	4,628

**Type of ED visit**	**2008**			**2009**			**2010**		
		
	**ED visits resulting in hospital admission**			**ED visits resulting in hospital admission**			**ED visits resulting in hospital admission**		
		
	**No.**		**(%)**	**No.**		**(%)**	**No.**		**(%)**

MHD-related visits	116,936		(35.7)	123,429		(34.1)	126,808		(31.1)
All other ED visits	580,655		(14.8)	597,177		(14.2)	619,831		(14.1)

**TABLE 2:** Mental health disorders (MHDs) resulting in emergency department (ED) visits and hospital admissions, by diagnostic category — North Carolina, 2008–2010

Type of MHD*	ICD-9-CM codes	% of MHD-related ED visits in this category†			Risk ratio for hospital admission§	Mean % admitted 2008–2010

		2008	2009	2010		
Stress/Anxiety/Depression	300 (excluding 300.9), 306, 308, 309, 311, 313.1, V11.2, V69.8, V79.0	60.78	61.70	62.33	0.91 (0.90–0.92)	28.89
Schizophrenia/Delusional/Psychosis	294.0, 294.8, 294.9, 295, 297, 298, V11.0	19.89	19.37	19.49	1.08 (1.07–1.09)	42.99
Bipolar	296, V11.1	17.96	18.26	18.32	1.28 (1.27–1.29)	37.32
Suicidal/Homicidal ideation	300.9, V62.84, V62.85	6.69	6.87	6.82	1.44 (1.42–1.45)	40.01
Dementia	290, 294.1, 294.2	5.99	5.53	5.21	1.26 (1.25–1.27)	60.54
Personality/Conduct disorder	301, 312	3.03	2.93	2.05	1.37 (1.35–1.39)	48.38
Miscellaneous/Other¶	302, 307 (excluding 307.1, 307.5, 307.8), V11.8, V11.9, V15.4 (excluding V15.41)	1.61	1.47	1.41	0.81 (0.79–0.83)	24.49
Psychiatric examination	V70.1, V70.2, V71.0	1.02	1.06	1.03	0.49 (0.47–0.52)	13.35
Mental disorders from brain damage	310	0.74	0.69	0.68	0.86 (0.83–0.89)	23.81
Developmental disorders originating in childhood	299	0.64	0.75	0.71	0.96 (0.91–1.01)	15.87
Eating disorders	307.1, 307.5	0.20	0.44	0.16	1.01 (0.95–1.06)	32.36

**Abbreviation:** ICD-9-CM = *International Classification of Diseases, Ninth Revision, Clinical Modification*.

* Up to 11 ICD-9-CM diagnostic codes were examined to classify presence or absence of categories of MHDs.

† Percentages in each column sum to more than 100% because 16% of MHD-related ED visits during 2008–2010 were counted in more than one MHD category.

§ Risk ratio for the presence of each condition versus its absence, controlling for number of diagnostic codes of any type (classified as either 6–11 codes or 1–5 codes), tobacco use, and presence or absence of nine comorbidities (substance abuse, injury, asthma/chronic obstructive pulmonary disorder, cancer, diabetes/hypoglycemia, heart failure, hepatic failure, renal failure, and obesity). Computed using log binomial regression with Poisson robust variances.

¶ Includes sexual and gender-identity disorders, personal history of other or unspecified mental disorder, personal history of psychiatric trauma, and special symptoms or syndromes not elsewhere classified.

**TABLE 3:** Risk for hospital admission after emergency department (ED) visits related to mental health disorders (MHDs) versus all ED visits, by age group — North Carolina, 2008–2010

Age group (yrs)	Risk ratio for hospital admission after an MHD-related ED visit*	% of MHD-related ED visits occurring in this age group	% of MHD-related ED visits in this age group resulting in hospital admission	% of all ED visits in this age group resulting in hospital admission
0–14	1.00 (referent)	2.30	14.03	3.73
15–24	1.22 (1.18–1.26)	10.99	17.70	4.70
25–44	1.36 (1.31–1.40)	31.12	22.19	7.84
45–64	1.79 (1.73–1.86)	28.33	36.52	20.01
≥65	2.21 (2.13–2.28)	27.25	51.19	38.76

* Computed using log binomial regression with Poisson robust variances, controlling for other MHDs, tobacco use, and presence or absence of nine comorbidities (substance abuse, injury, asthma/chronic obstructive pulmonary disorder, cancer, diabetes/hypoglycemia, heart failure, hepatic failure, renal failure, and obesity).

**TABLE 4:** Population-based rates* of emergency department (ED) visits related to mental health disorders (MHDs), by diagnostic category, age group, and year — North Carolina, 2008–2010

Age group and year	Diagnostic category†											

	Any MHD diagnosis (all categories combined)	Stress/Anxiety/Depression	Schizophrenia/Delusional/Psychosis	Bipolar	Suicidal/Homicidal ideation	Dementia	Personality/Conduct disorder	Miscellaneous/Other	Psychiatric examination	Mental disorders from brain damage	Developmental disorders originating in childhood	Eating disorders
**0–14 yrs**												
2008	43.7	15.5	1.7	8.3	2.8	0.1	4.1	1.7	1.4	1.0	6.8	0.3
2009	50.2	16.2	1.9	8.4	3.4	0.2	4.2	1.8	1.1	1.1	8.8	3.1
2010	48.1	16.8	1.9	8.8	3.5	0.2	4.4	1.8	1.2	1.3	7.8	0.4
**15–24 yrs**												
2008	288.3	170.8	18.5	57.0	17.4	0.4	7.7	4.0	4.9	3.5	3.2	0.7
2009	316.6	183.9	18.1	66.6	20.1	0.3	8.2	4.4	5.5	4.0	3.8	1.7
2010	331.3	192.1	20.7	68.3	22.7	0.2	8.8	4.0	5.5	3.9	4.2	0.8
**25–44 yrs**												
2008	415.4	260.8	32.4	87.4	18.1	0.2	4.9	3.8	4.0	2.6	0.7	0.6
2009	455.4	288.2	31.8	95.2	21.0	0.4	5.5	4.1	4.1	2.8	1.1	1.3
2010	482.0	308.1	34.2	97.5	23.5	0.3	5.6	4.2	4.0	3.0	1.2	0.5
**45–64 yrs**												
2008	410.8	267.1	48.2	66.6	12.5	3.4	3.8	3.7	3.2	1.9	0.3	0.3
2009	451.0	296.9	50.9	71.2	14.8	3.7	3.9	3.5	3.2	2.0	0.3	0.7
2010	483.0	318.1	52.6	77.1	17.6	4.0	3.8	4.5	3.1	2.0	0.3	0.3
**≥65 yrs**												
2008	840.4	308.2	321.0	34.0	3.2	158.5	2.2	6.5	1.4	4.6	0.0	0.6
2009	865.3	324.0	336.1	34.1	4.0	152.5	2.2	6.0	1.6	3.7	0.1	1.1
2010	905.8	344.1	355.7	35.4	5.4	150.5	2.3	8.0	1.6	3.8	0.1	0.3

* Per 10,000 population.

† Diagnostic category for each MHD-related ED visit based on the category of the first-listed MHD *International Classification of Diseases, Ninth Revision, Clinical Modification* code.
